# Supplementary material for: Child’s disability status and postnatal healthcare utilization among forcibly displaced women in Pakistan: A Secondary data analysis
Source: PLOS Glob Public Health. 2026 Jan 27;6(1):e0005795. doi: 10.1371/journal.pgph.0005795 (PMC12843506; doi:10.1371/journal.pgph.0005795)
Supplement: Attachment — Submitted filename: Response to Reviewers.docx.pdf [file pgph.0005795.s002.pdf]

Reviewer #1: Review of "Child's disability status and postnatal healthcare utilization among forcibly displaced women: Evidence from Pakistan"
Date: 18/10/2025

Overall Comment

This is a good effort overall, and I appreciate the attempt to use national data to explore an important topic. However, the study's framing limits its relevance and interpretive value. The current direction of the analysis, treating impairment as the exposure and postnatal care (PNC) uptake as the outcome, feels counterintuitive and does not add much beyond what could be speculated without data. It may be more meaningful to reverse the relationship, using PNC as the exposure variable. This would allow the study to explore whether PNC contributes to early diagnosis of neonatal impairment or, among children beyond the neonatal period, whether PNC influences the likelihood of disability. Additionally, the analytic process and sample selection are unclear, particularly how the author moved from the overall sample of 8,264 respondents to the subset used for analysis.

We thank the reviewer for this thoughtful comment and appreciate the opportunity to clarify the conceptual framing of our analysis. We respectfully disagree that treating impairment as the exposure and PNC uptake as the outcome limits interpretive value. Our decision to model impairment as the exposure was grounded in both theoretical and empirical rationale consistent with prior public health and disability research.

First, from **a** social determinant of health perspective, impairment or disability status represents a key *structural and biological exposure* that can influence healthcare access and utilization. Understanding how physical or sensory impairment affects postnatal care uptake is essential for identifying barriers within health systems that disproportionately affect women with children with disabilities.

Second, the directionality of our analysis reflects real-world association rather than speculation. Impairment in women or their children often precedes and shapes a woman's interaction with the healthcare system, affecting mobility, access to information, autonomy, and provider attitudes. Modeling impairment as the exposure allows us to quantify these relationships and generate evidence-based insights for inclusive maternal and child health interventions.

Third, our approach contributes empirical value by moving beyond assumptions. While qualitative and policy literature often speculate that women with impairments face greater challenges in accessing care, there is limited quantitative evidence quantifying the magnitude and determinants of this disparity in low-resource settings, particularly among the displaced population. Our findings thus offer novel data-driven insights that extend beyond anecdotal understanding.

That said, in response to the reviewer's concern, we have revised the Introduction and methods to articulate this conceptual logic better and to explicitly acknowledge the theoretical basis for treating impairment as an exposure variable. We Added this text to the introduction:

Thus, this study aimed to explore the association between a child's disability status and postnatal healthcare utilization among forcibly displaced women in Pakistan, and to identify the socioeconomic and health-system factors that influence postnatal care use within this population. We conceptualize impairment as an exposure variable influencing postnatal care (PNC) utilization. This framing aligns with the social model of disability and the World Health Organization's International Classification of Functioning, Disability and Health (ICF), which views impairment as a structural determinant that shapes access to health services. Women with children with physical, sensory, or cognitive impairments often face systemic, logistical, and attitudinal barriers that can limit their ability to seek and receive timely PNC. By examining impairment as the exposure, we aim to quantify its influence on PNC uptake and identify the health system and social barriers that contribute to inequities in postnatal service use among women with children with disabilities (see page 5)

Additionally, we have included the sentence below in the methods section.

The primary independent variable was child impairment status, defined in accordance with the World Health Organization's International Classification of Functioning, Disability and Health (ICF) framework. In this study, *impairment* refers to any physical, sensory, or cognitive limitation in a child as reported by the mother at the time of the survey. Mothers were asked whether any of their children had a condition or limitation that affected movement, sight, hearing, speech, learning, or behavior. Because the HAUS survey did not restrict the timing of the impairment's onset, reported cases may reflect conditions recognized at birth or during early infancy. Conceptually, we treated impairment as the exposure variable since it represents a child-level factor that can shape maternal healthcare-seeking behavior, particularly postnatal care utilization. This framing aligns with the social model of disability, which recognizes impairment as a determinant of access and interaction within health systems, rather than as a health outcome. Treating impairment as an exposure, therefore, enables a data-driven assessment of how disability status translates into differential access to essential maternal health services. This perspective advances the literature by moving beyond descriptive accounts of vulnerability to provide empirical evidence on the extent and determinants of inequities in PNC utilization among women with children with disabilities in resource-limited settings. (see page 8)

Here are more detailed comments for each section:

Title

2. It should be specified in the title that this is a secondary data analysis. Also would be good to include the timeframe in the title

We thank the reviewer for this comment. We changed the title to read:

Child's Disability Status and Postnatal Healthcare Utilization Among Forcibly Displaced Women in Pakistan: A Secondary Data Analysis (see page 1)

Abstract

3. Methods: Start from the study type – it is a secondary data analysis. Also missing the sample size, time frame, inclusion criteria (e.g. mothers who gave birth in the past year?). Please clarify what your outcome variable is. I cant tell if its disability status, PNC utilization or even both. "…..Postnatal care (OR= 0.9.99, CI= 3.32-30.05)" The OR here seems wrong… did you mean 9.99? Also, it would be good to add p values, although the CIs hint at the significance

The direction of association for tertiary education seems counterintuitive. The authors should interpret why higher education correlates with lower use of care or verify if it's a misclassification.

Conclusion: The conclusion repeats general awareness messaging rather than specific interpretations and recommendations from their study. Recommendations should directly follow from the statistical findings (e.g. targeting the educational disparities you found or advocating for continuity of ANC-PNC use etc).

We appreciate this valuable feedback and have revised the manuscript to clarify the study design, timeframe, inclusion criteria, and outcome variable. Specifically, we have revised the Abstract to include these clarifications for greater transparency.

The abstract has been revised to read:

Background: The World Health Organization (WHO) estimates that about half of maternal and 40% of neonatal deaths occur within 24 hours of childbirth. Many women and newborns, especially among forcibly displaced populations, lack timely access to essential care. While research has examined antenatal and postnatal care (PNC) utilization in low-resource settings, little is known about displaced women raising children with disabilities. This study explores factors influencing PNC utilization among forcibly displaced women in Pakistan, focusing on displacement, maternal health, and childhood impairment.

Methods: Using secondary data from the 2022 UNHCR Health and Utilization Survey (HAUS), this study examined the relationship between a child's disability and maternal PNC utilization among 2,847 displaced mothers aged 18–49 who had given birth within the past year. Multivariable logistic regression assessed how child impairment influenced PNC use while controlling for socioeconomic, demographic, and healthcare access factors.

Results: Among respondents, 65% utilized PNC services, and 17.6% reported having a child with an impairment. Most were married (85.9%), had completed primary education (88.9%), and delivered in public health facilities. Two-thirds had at least one antenatal care (ANC) visit. Child impairment significantly influenced maternal PNC utilization (OR = 1.24; 95% CI: 1.02–1.76). Mothers with tertiary education and children with impairments were less likely to use PNC

compared with those with primary education (OR = 0.21; 95% CI: 0.09–0.52). ANC strongly predicted PNC use (OR = 9.99; 95% CI: 3.32–30.05). Other significant predictors included place of delivery, healthcare authorization, and ability to pay.

Conclusion: Women who accessed antenatal care were nearly ten times more likely to use postnatal services. Findings highlight the need to strengthen ANC–PNC continuity and address financial, structural, and legal barriers limiting equitable, disability- and displacement-sensitive maternal healthcare in Pakistan. (see page 2)

We also added this sentence to the discussion:

Although several studies [19, 20] argue that educational achievement is connected to postnatal care and healthcare system utilization. Interestingly, our results indicate that women with tertiary education were less likely to utilize PNC services compared to those with only primary education. Although this appears counterintuitive, the result may reflect the complex interplay between structural barriers and displacement-related constraints rather than education itself. Forcibly displaced women with higher education often face employment restrictions, limited autonomy, or administrative barriers in host-country health systems that constrain healthcare access. This finding aligns with research suggesting that education alone does not translate into health service use when socioeconomic, legal, or geographic obstacles persist in humanitarian settings. (see pages 19)

Background
1. Be consistent with the bracket types used for citations
2. Add a clear objective statement at the end of the introduction for easy accessibility

We thank the reviewer for this comment. We concluded the following statement to the introduction:

Therefore, this study aimed to examine the association between a child's disability status and postnatal healthcare utilization among forcibly displaced women in Pakistan, and to identify the socioeconomic and health-system factors that influence postnatal care use within this population.

Methods
Setting
1. "Pakistan currently faces one of the longest-running refugee crises in the world, with

over 1.3 million Afghan refugees officially registered within its borders" ... this sentence should be referenced

A reference has been added to the sentence, which now reads:

Pakistan currently faces one of the longest-running refugee crises in the world, with over 1.75 million Afghan refugees officially registered within its borders (*United Nations High Commissioner for Refugees [UNHCR], 2025*).

2. More info is needed to be able to assess the representativeness of the HAUS dataset. Either you include a reference to the database, where it is properly described, or you give your readers a sense of the geographic coverage of the survey.

3. The authors should include when the data was collected and the sample scope

4. Since this study focuses on PNC, this section should also focus on neonatal health and PNC services among this population

We thank the reviewer for this helpful suggestion. We have revised the *Study Setting and Data Source* section to include a description of the HAUS dataset's geographic coverage, population scope, and sampling approach, as well as a formal reference to the publicly available UNHCR microdata repository.

Pakistan hosts one of the world's longest-standing refugee populations, with over 1.75 million Afghan refugees officially registered within its borders (United Nations High Commissioner for Refugees [UNHCR], 2025). Although current national policies allow refugees to access the public health system on the same terms as Pakistani citizens, disparities persist in the quality and continuity of maternal and newborn care. Evidence suggests that refugee women face significant barriers to accessing antenatal and postnatal care (PNC) services, including financial constraints, limited mobility, and inadequate awareness of available health resources. These barriers are particularly concerning given the high rates of neonatal morbidity and mortality within displaced communities, where complications during the immediate postpartum period often go undetected or untreated. The absence of standardized data on refugee maternal and child health outcomes has further hindered efforts to evaluate PNC service coverage, identify service gaps, and develop targeted interventions to improve neonatal survival and maternal well-being among forcibly displaced populations in Pakistan.

To address this gap, the United Nations High Commissioner for Refugees (UNHCR) implemented the Health and Utilization Survey (HAUS), .a nationally representative survey conducted in 2022 across major Afghan refugee-hosting areas in Pakistan. Implemented in collaboration with the Government of Pakistan's Commissionerate for Afghan Refugees (CAR**)** and the World Bank**,** the survey covered refugee settlements and urban host communities in Khyber Pakhtunkhwa, Balochistan, and Punjab, the three provinces hosting over 90% of registered Afghan refugees. HAUS employed a stratified sampling approach to ensure representativeness by province, settlement type, and household composition. Data collection included demographics, healthcare access and utilization, barriers, and maternal and child health outcomes. The dataset includes over 8,000 refugee households, making it one of the most comprehensive sources on refugee health in Pakistan. Full documentation, including sampling methodology and weighting procedures, is available via the UNHCR Microdata Library. (see pages 5-6)

Dependent Variable

5. First include the definition of PNC here, so your readers are sure that it is the same as the global standards.

Dependent variable

Thank you for this suggestion. We added this to the manuscript:

We have revised the *Dependent Variable* section to include the WHO's definition of postnatal care (PNC), clarifying that our operationalization aligns with global standards.

The dependent variable for this study was postnatal care (PNC) utilization**.** According to the World Health Organization (WHO, 2013**)**, the postnatal period refers to the six weeks following childbirth, a critical phase for the health and survival of both mother and newborn. Postnatal care includes the assessment of maternal and neonatal health, detection and management of complications, counseling on breastfeeding, family planning, and hygiene, as well as immunization services**.** In the HAUS dataset, PNC utilization was measured based on the question: *"Did you receive postnatal care services following your most recent delivery?"* Responses were coded dichotomously as **Yes (1)** for women who reported receiving PNC and **No (0)** for those who did not. This operational definition aligns with WHO's global maternal health monitoring

framework and ensures comparability with other studies in low- and middle-income country contexts. (See page 8)

Primary independent variable

6. Please clarify what you mean by impairment, as most disabilities are not typically apparent during the neonatal period. From your description, it appears that you used data based on mothers' reports of whether their child had a disability during the neonatal period, and then analyzed this in relation to postnatal care (PNC) uptake. However, conceptually, it seems more appropriate for PNC uptake to serve as the independent variable in this relationship.

We thank the reviewer for this insightful comment. We acknowledge that impairments are not always identified immediately after birth and may become apparent within the early postnatal period. In the HAUS dataset, impairment refers to any physical, sensory, or cognitive limitation in a child as perceived and reported by the mother at the time of the survey, regardless of whether it was detected at birth or in the months following.

We recognize the reviewer's conceptual point; however, our framing treats impairment as the exposure variable because it represents a child-level condition that precedes and influences maternal health-seeking behavior, rather than being a consequence of PNC use. This approach is consistent with **the** social model of disability **and** WHO's *International Classification of Functioning, Disability and Health (ICF)* framework, which conceptualize impairment as a structural determinant that affects interaction with the health system.

To enhance clarity, we have revised the *Methods* section to define "impairment" precisely and to explicitly justify the analytical direction of our model.

The primary independent variable was child impairment status, defined in accordance with the World Health Organization's International Classification of Functioning, Disability and Health (ICF) framework. In this study, *impairment* refers to any physical, sensory, or cognitive limitation in a child as reported by the mother at the time of the survey. Mothers were asked whether any of their children had a condition or limitation that affected movement, sight, hearing, speech, learning, or behavior. Because the HAUS survey did not restrict the timing of the impairment's onset, reported cases may reflect conditions recognized at birth or during early infancy. Conceptually, we treated impairment as the exposure variable since it represents a child-level factor that can shape maternal healthcare-seeking behavior, particularly postnatal care utilization. This framing aligns with the social model of disability, which recognizes impairment as a determinant of access and interaction within health systems, rather than as a health outcome. Treating impairment as an exposure, thus, enables a data-driven assessment of how disability status translates into differential access to essential maternal health services. This perspective advances the literature by moving beyond descriptive accounts of vulnerability to provide empirical evidence on the extent and determinants of inequities in PNC utilization among women with children with disabilities in resource-limited settings. (see page 8)

Statistical analysis

7. You should always include the company and place when mentioning statistical software - Stata version 18 (StataCorp LLC, College Station, TX, USA)

Thank you for bringing this to our attention. This has been added to the manuscript

8. Was there missing data? And if yes, how did you treat it?

Thank you for this important question. We have now included a description of how missing data were assessed and treated in the *Data Analysis* section. We confirm that missingness was minimal across variables, and complete case analysis was used after confirming that the proportion of missing data did not exceed 5% for any variable.

Prior to analysis, all variables were assessed for missingness and internal consistency. The proportion of missing data across variables was generally low (<5%) and was therefore handled through listwise deletion (complete case analysis). Since the overall level of missingness was minimal, no imputation procedures were applied. This approach is consistent with best practices for secondary data analyses using population-based surveys where missing data are sparse and unlikely to bias estimates

Results

1. Authors should start by telling us the number of participants

A total of 2847 forcibly displaced women aged 18–49 years were included in this analysis.

2. The sample description and analytical pathway are quite unclear. It is difficult to understand how the sample was derived and how it aligns with the study objectives.

• The total sample size of 8,264 respondents appears to include both men and women, but the study's focus (postnatal care utilization among parents of children with impairments) suggests that only a specific subset, like women of reproductive age or those who recently gave birth (within a specified period, should have been analyzed. You need to clearly define who was included in this analysis and why.

• Please specify the inclusion criteria, for example: "Women who gave birth within the last 12 months" or "Mothers of children aged under five."

Then, state the sample size for that subset and use that as your analytical sample, not the entire dataset.

• It is not clear how the analysis moved from the initial 8,264 respondents to the smaller numbers used in regression. You need to explain any exclusions or missing data and show the flow of the sample (perhaps in a short paragraph or figure).
• Without clarity on the sample used, the validity of the results especially the odds ratios and discussion, cannot be properly assessed.

Thank you for this important observation. We have clarified the composition of our analytic sample and the inclusion criteria. Although the full HAUS dataset includes both male and female respondents, our analysis was restricted to women of reproductive age (18–49 years) who had given birth within 12 months preceding the survey. This subset was selected because postnatal care (PNC) utilization is specifically relevant to women who have recently experienced childbirth.

We added this to the manuscript:

This study used data from the United Nations High Commissioner for Refugees (UNHCR) Health and Access Utilization Survey (HAUS)**.** The Health and Access Utilization Survey (HAUS) dataset includes data from both male and female respondents across refugee households. For this analysis, we restricted the sample to women of reproductive age (18–49 years) who had given birth within the 12 months preceding the survey, aligning with the World Health Organization's definition of the postnatal period. Respondents without recent childbirth or with missing data on key variables were excluded. The final analytic sample consisted of 2,847 forcibly displaced women. Male respondents and women without recent childbirth were excluded from the analysis to ensure conceptual and analytical consistency with the study's objective of examining postnatal healthcare use among mothers raising children with impairments. (See page 9)

3. In summary, please:
• Clearly define your study population.
• Report the final sample size after applying inclusion/exclusion criteria.
• Ensure that only the relevant subgroup (e.g., mothers who recently gave birth) is analyzed.
• Provide a short explanation of how the data were cleaned or filtered before analysis.
• Include these in the methodology section

We thank the reviewer for the above suggestions. We address all these points

7. In addition to AIC and BIC, please include model fit statistics such as the Hosmer–

Lemeshow goodness-of-fit test and pseudo R^2 values (e.g., Cox–Snell or Nagelkerke). These help assess how well the logistic regression model explains and fits the data.

We thank the reviewer for this important suggestion. We have now included the Hosmer–Lemeshow goodness-of-fit test and pseudo R^2 values (Cox–Snell and Nagelkerke**)** to evaluate model fit and explanatory power. These statistics are now reported in the *Data Analysis* subsection and summarized at the end of the *Results* section. Both the Hosmer–Lemeshow test and pseudo R^2 values indicate that the final multivariable model fits the data adequately.

And added this to the results:

The final multivariable logistic regression model demonstrated a satisfactory fit, with a **Hosmer–**Lemeshow chi-square of 7.82 and a p-value of 0.45**,** indicating no evidence of poor fit. The model explained a meaningful portion of the variance in postnatal care utilization (Cox–Snell $R^2 = 0.18$; Nagelkerke $R^2 = 0.27$). AIC and BIC values were 620 and 889, respectively, further confirming the parsimony of the final model. (see page 13)

8. It remains unclear how "child impairment" was defined or measured. This is crucial for interpreting results and understanding the validity of your results.

This has been addressed in the above responses.

Discussion

1. The central finding in this study, that mothers of impaired children were more likely to use PNC, is not adequately explained. The discussion speculates on emotional motivation (overwhelmed mothers), which I think is a week argument. The authors should consider alternative explanations like health complications prompting more follow-up visits, mandatory health checks for infants with visible conditions, or even reverse causality if impairment was diagnosed during a postnatal visit.

The discussion has been modified to read:

Our results show that mothers whose children had some form of impairment after birth were more likely to utilize postnatal care (PNC) services, which contrasts with the common assumption that disability is associated with lower healthcare access. Mothers of children with impairments often recognize that their child requires more medical attention, monitoring, or specialized care. This awareness motivates them to seek postnatal checkups and follow-up services more diligently, ensuring the child's condition is evaluated, managed, or referred appropriately. Beyond that,

several explanations may account for this finding. First, infants with visible or suspected impairments are often flagged for follow-up care by healthcare providers, prompting mothers to return for postnatal or pediatric visits. Second, it is possible that some impairments were identified during postnatal consultations, resulting in a positive association between impairment and PNC use due to reverse causality rather than a direct causal effect. Third, mothers of infants with early health complications or developmental concerns may receive mandatory or advised health checks from providers, increasing their likelihood of PNC attendance. In addition, although most forcibly displaced people require authorization from host governments to access healthcare, our findings suggest that parents often prioritize their children's health needs by engaging in postnatal services when possible. Research indicates that, despite unmet needs, children with disabilities are generally more likely to seek healthcare compared to their peers without disabilities [12]. This suggests a broader pattern of increased health system engagement among families facing health vulnerabilities. Moreover, postnatal care services, including genetic counseling, immunization, and growth monitoring, play a critical role in the primary prevention of childhood disabilities [13]. Taken together, these mechanisms suggest that the observed association may partly reflect the health system's responsiveness to neonatal complications rather than a purely behavioral response by mothers. Nonetheless, the findings underscore that PNC encounters are essential opportunities for early support for children with impairments in humanitarian contexts. Future longitudinal and qualitative studies are needed to clarify the temporal and causal relationship between child impairment, diagnosis, and healthcare utilization among forcibly displaced women. (See page 18)

2. The manuscript still reads as if child impairment precedes postnatal care utilization. I think this conceptually problematic, since most impairments are not detected immediately at birth. The authors need to clarify whether impairment was identified postnatally and, if so, reframe the interpretation to reflect that PNC may predict impairment identification, not the other way around.

We acknowledge that our earlier framing may appear to assume that impairment precedes postnatal care utilization. To clarify, our theoretical model is grounded in the social determinants of health perspective, which conceptualizes impairment as a structural condition that shapes healthcare access and utilization. However, given **the** cross-sectional nature of the HAUS dataset and the fact that the timing of impairment identification is not specified, it is also plausible that some impairments were recognized during postnatal visits, suggesting potential reverse temporality in certain cases.

We therefore interpret our findings cautiously, noting that while the analysis is theoretically framed with impairment as an exposure, the association observed may also reflect postnatal care functioning as a platform for early detection of impairments**.** This balanced interpretation aligns theoretical rigor with empirical transparency and strengthens the validity of our conclusions.

We added this to the limitation section of the manuscript

Our results show that mothers of children reported to have impairments were more likely to utilize postnatal care (PNC) services. However, because impairments were identified based on maternal reports at the time of the survey, it is not possible to determine whether these impairments were present at birth or recognized later. It is therefore plausible that the association reflects **reverse temporality**, where PNC use facilitates the **identification or diagnosis of impairments** rather than the impairment motivating PNC utilization.

3. The discussion cites studies from Uganda, Ethiopia, and Nepal but does not connect the findings to humanitarian contexts, which was the paper's stated contribution to the field. It should highlight how displacement, legal status, and healthcare authorization shape maternal care or PNC utilization patterns.

We thank the reviewer for this insightful comment. We have revised the *Discussion* to explicitly situate our findings within the humanitarian and displacement context. Specifically, we now discuss how displacement-related vulnerabilities, **including** legal restrictions, limited healthcare authorization, mobility constraints, and dependency on host-country policies**,** shape women's access to and use of postnatal care. This addition strengthens the paper's contribution to understanding maternal healthcare utilization in refugee settings.

Our findings must be interpreted within the broader realities **of** humanitarian displacement**,** where legal status and healthcare authorization fundamentally shape maternal healthcare access. In Pakistan, Afghan refugees and other forcibly displaced women are required to obtain formal authorization to access national health facilities, which can delay or discourage healthcare-seeking, particularly during the postnatal period. **M**obility restrictions, dependency on host-country health system**s**, and the absence of consistent health insurance coverage compound this barrier. Women who secure authorization or pay for private delivery services are more likely to access PNC, suggesting that legal and financial gatekeeping mechanisms play a decisive role in determining care continuity after childbirth.

Unlike women in stable low-resource communities, displaced mothers navigate complex institutional pathways where healthcare access is conditional on administrative approval or donor-funded programs**.** This structural dependency distinguishes humanitarian populations from other LMIC contexts and may explain the observed disparities in PNC utilization. Our results therefore contribute to the growing literature emphasizing that humanitarian health inequities are produced not only by poverty or education, but also by governance structures and policy regimes that regulate refugee health rights and entitlements. Addressing these barriers requires policy

coordination between host governments, UNHCR, and development partners to ensure that maternal and postnatal services are accessible regardless of legal status.

Conclusion

1. This is way too long. It should highlight the key findings, the implications and the key recommendations, written succinctly.

We thank the reviewer for this comment. The conclusion has been revised to read:

The study has notable limitations. Besides the inherent issues with secondary data, it relied on questions about past activities, which increases the risk of recall bias. There is also a possibility of selection bias, as individuals negatively affected by a lack of healthcare access may be more inclined to participate than others. Additionally, since the data is self-reported, it may be subject to biases such as overreporting or underreporting. Our results show that mothers of children reported to have impairments were more likely to utilize postnatal care (PNC) services. However, because impairments were identified based on maternal reports at the time of the survey, it is not possible to determine whether these impairments were present at birth or recognized later. It is therefore plausible that the association reflects reverse temporality, where PNC use facilitates the identification or diagnosis of impairments rather than the impairment motivating PNC utilization. (See page

Reviewer #2: Comments

1. Primary Independent Variable: Child impairment: Lacks operational definition, what threshold determines impairment?

We thank the reviewer for bringing this vital point to our attention. We have operationalized our primary independent variable. It now reads:

The primary independent variable was child impairment status, defined in accordance with the World Health Organization's International Classification of Functioning, Disability and Health (ICF) framework. In this study, *impairment* refers to any physical, sensory, or cognitive limitation in a child as reported by the mother at the time of the survey. Mothers were asked whether any of their children had a condition or limitation that affected movement, sight, hearing, speech,

learning, or behavior. Because the HAUS survey did not restrict the timing of the impairment's onset, reported cases may reflect conditions recognized at birth or during early infancy. Conceptually, we treated impairment as the exposure variable since it represents a child-level factor that can shape maternal healthcare-seeking behavior, particularly postnatal care utilization. This framing aligns with the social model of disability, which recognizes impairment as a determinant of access and interaction within health systems, rather than as a health outcome. Treating impairment as an exposure, thus, enables a data-driven assessment of how disability status translates into differential access to essential maternal health services. This perspective advances the literature by moving beyond descriptive accounts of vulnerability to provide empirical evidence on the extent and determinants of inequities in PNC utilization among women with children with disabilities in resource-limited settings.

2. Dependent Variable definition: "Since delivery" is vague, no timeframe specified (within 48 hours? 6 weeks?)
3. No description of what constitutes PNC services (checkup only? Multiple visits? Specific components?)

We have revised the *Dependent Variable* section to include the WHO's definition of postnatal care (PNC), clarifying that our operationalization aligns with global standards.

The dependent variable for this study was postnatal care (PNC) utilization**.** According to the World Health Organization (WHO, 2013**)**, the postnatal period refers to the six weeks following childbirth, a critical phase for the health and survival of both mother and newborn. Postnatal care includes the assessment of maternal and neonatal health, detection and management of complications, counseling on breastfeeding, family planning, and hygiene, as well as immunization services**.** In the HAUS dataset, PNC utilization was measured based on the question: *"Did you receive postnatal care services following your most recent delivery?"* Responses were coded dichotomously as **Yes (1)** for women who reported receiving PNC and **No (0)** for those who did not. This operational definition aligns with WHO's global maternal health monitoring framework and ensures comparability with other studies in low- and middle-income country contexts.

Recommendations:
1. Specify the impairment assessment criteria
2. Report types and distribution of impairments (physical, cognitive, sensory)

3. Define the specific timeframe for PNC (e.g., "within 42 days postpartum")
4. Describe what services were included in the PNC definition

1) “Specify the impairment assessment criteria”

The primary independent variable was child impairment status, defined in accordance with the World Health Organization’s International Classification of Functioning, Disability and Health (ICF) framework. In this study, *impairment* refers to any physical, sensory, or cognitive limitation in a child as reported by the mother at the time of the survey. Mothers were asked whether any of their children had a condition or limitation that affected movement, sight, hearing, speech, learning, or behavior. Because the HAUS survey did not restrict the timing of the impairment’s onset, reported cases may reflect conditions recognized at birth or during early infancy. Conceptually, we treated impairment as the exposure variable since it represents a child-level factor that can shape maternal healthcare-seeking behavior, particularly postnatal care utilization. This framing aligns with the social model of disability, which recognizes impairment as a determinant of access and interaction within health systems, rather than as a health outcome. Treating impairment as an exposure, thus, enables a data-driven assessment of how disability status translates into differential access to essential maternal health services. This perspective advances the literature by moving beyond descriptive accounts of vulnerability to provide empirical evidence on the extent and determinants of inequities in PNC utilization among women with children with disabilities in resource-limited settings.

2) “Report types and distribution of impairments (physical, cognitive, sensory)”

HAUS did not organize distributions for these impairments. They asked participants if their child had any physical, cognitive, or sensory impairments and provided examples. However, any 'yes' response resulted in classification as an impairment.

3) “Define the specific timeframe for PNC (e.g., 'within 42 days postpartum')”

4) “Describe what services were included in the PNC definition”

The dependent variable for this study was postnatal care (PNC) utilization**.** According to the World Health Organization (WHO, 2013**)**, the postnatal period refers to the six weeks following childbirth, a critical phase for the health and survival of both mother and newborn. Postnatal care includes the assessment of maternal and neonatal health, detection and management of complications, counseling on breastfeeding, family planning, and hygiene, as well as immunization services**.** In the HAUS dataset, PNC utilization was measured based on the question:

"Did you receive postnatal care services following your most recent delivery?" Responses were coded dichotomously as **Yes (1)** for women who reported receiving PNC and **No (0)** for those who did not. This operational definition aligns with WHO's global maternal health monitoring framework and ensures comparability with other studies in low- and middle-income country contexts.

.
